# Phalaenopsis pollinia storage at sub-zero temperature and its pollen viability assessment

**DOI:** 10.1186/s40529-017-0218-2

**Published:** 2018-01-03

**Authors:** Shih-Chang Yuan, Shih-Wen Chin, Chen-Yu Lee, Fure-Chyi Chen

**Affiliations:** Department of Plant Industry, National Pingtung University of Science & Technology, Pingtung, 91201 Taiwan

**Keywords:** Low temperature storage, Pollen viability, In vitro germination, Hand pollination

## Abstract

**Background:**

In a breeding program, usually only superior parents are chosen for cross hybridization. Pollens of elite cultivars may not be available at hand. Properly stored pollens provide an opportunity for cross hybridization at unavailable time.

**Results:**

Pollen of a *Phalaenopsis* hybrid was evaluated for the storage ability at different temperatures, including room temperature, 4, − 20, and − 80 °C for up to 96 weeks. The viability of pollen was assessed by TTC staining, in vitro germination and hand pollination during and after storage. Pollen stored at all temperatures for 4 weeks remained viable and capable of successful pollination. Pollen lost its viability after 4 weeks at room temperature. Pollen remains viable after 40 weeks at 4 °C, and after 96 weeks at both − 20 and − 80 °C of storage. Viable pollen could be successfully pollinated to the female parent at all effective storage conditions and produced seeds.

**Conclusions:**

Our results indicate that *Phalaenopsis* pollen can be stored at 4 °C up to 40 weeks for short-term purpose. For long-term storage, pollen can be kept at both − 20 and −80 °C.

## Background

Phalaenopsis is one of the economically important orchids for potted plant and cut flower production worldwide. There are about 60 species of the *Phalaenopsis* genus which are native to tropical Asia and the larger islands of the Pacific Ocean, where they ranged from Sri Lanka and South India in western to Papua New Guinea in eastern and extended to southern China, Taiwan, and the north Philippines (Sweet 1980; Christenson 2001). In Taiwan, the potted plants of *Phalaenopsis* have been exported in large quantity annually during 2001–2016 (Council of Agriculture 2016), partly due to the continuous development of novel varieties. In order to continuously supply enough quantity of young plants to the market, micropropagation of newly selected cultivars from cross-hybridization program is demanded at all times. In a breeding program, usually only superior parents are chosen for cross-hybridization (Ajeeshkumar and Decruse 2013). However, pollens of elite cultivars may not be available at hand, therefore properly stored pollen becomes an alternative to keep the desirable germplasm for cross-pollination at need in plant improvement programs (Metz et al. 2000; Martínez-Gómez et al. 2002; Deng and Harbaugh 2004; Lora et al. 2006; Masum Akond et al. 2012; de Souza et al. 2014; Peng et al. 2015; Wang et al. 2015).

Pollen longevity or viability is defined as the stored pollen retaining its viability after long-term preservation (Vaknin and Disikowitch 2000). Several reports have shown that pollen stored at low temperatures was effective for long-term preservation, such as almond (Martínez-Gómez et al. 2002), cherimoya (Lora et al. 2006), crape myrtle (Masum Akond et al. 2012), Arabidopsis (Bou Daher et al. 2009), bromeliads (Parton et al. 2002; de Souza et al. 2014), pecan (Peng et al. 2015), caladium (Deng and Harbaugh 2004), jojoba (Vaknin et al. 2003), mango (Dutta et al. 2013) and lily (Wang et al. 2004). Pritchard and Prendergast (1989) used several orchid species to evaluate the pollen storage ability, including *Anacamptis pyramidalis*, *Cymbidium elegans*, *C. tracyanum*, *Dactylorhiza fuchsii*, *D. maculata*, *Epipactis purpurata*, *Gymnadenia conopsea*, *Listera ovata*, *Orchis mascula*, *O. morio*, and *Spiranthes spiralis*. The result indicated that pollen stored at both − 20 and − 196 °C offer the potential to extend its longevity up to at least 1 year. The storage conditions, including temperatures and relative humidity (RH) affect the viability of pollen (van der Walt and Littlejohn 1996; Deng and Harbaugh 2004). Besides, pollen age, flower physiological state as well as pollen moisture content also affect pollen viability during storage (Rosell et al. 2006; Soares et al. 2008).

The evaluation of pollen viability can be performed by use of fluorescein diacetate (FDA) (Heslop-Harrison and Heslop-Harrison 1970), fluorochromatic dye (FCR) (van der Walt and Littlejohn 1996), lactophenol cotton blue (LPCB) (Bellusci et al. 2010), 2,3,5-triphenyltetrazolium chloride (TTC) (Matison et al. 1947; Khatun and Flowers 1995; Sorkheh et al. 2011; Abdelgadir et al. 2012), or thiazolyl blue (MTT) staining (Khatun and Flowers 1995). Another way to examine the pollen viability was by in vitro germination using Brewbaker and Kwack medium (Brewbaker and Kwack 1963) and subsequently stained by Alexander’s dye (Alexander 1969; Galleta 1983). Although in vitro pollen germination is handy for determining its viability, it may not reveal the actual potential of pollination ability. Hand pollination probably is the best and easier way to estimate the effectiveness of pollen viability after storage (Lyakh et al. 1998).

Currently, no report is available on the cold storage of *Phalaenopsis* pollen. The objective of this study was to investigate appropriate temperature parameters for both short-term and long-term storage.

## Materials and methods

### Pollen source and storage condition

**Fig. 1:**
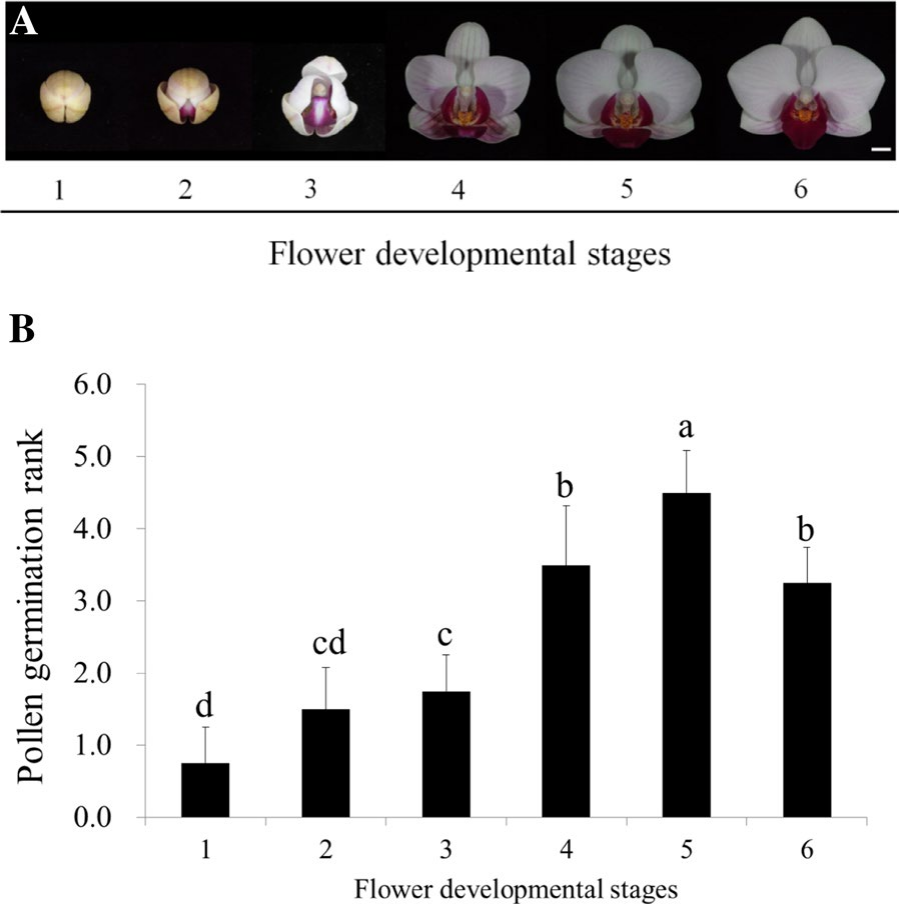
Flower developmental stages (**A**) and their corresponding pollen germination rate (**B**) in *Phalaenopsis* Little Gem Stripes. **A** Flower developmental stages are defined as: (1) tight floral bud; (2) sepal half-open; (3) flower half-open; (4) prior to anthesis; (5) flower fully open; (6) 1 day after fully open. **B** In vitro pollen germination of *P.* Little Gem Stripes was evaluated by incubating in Brewbaker and Kwack medium for 7 days at 25 ± 1 °C and then by Alexander staining. Bars represent mean ± SE, n = 4. Pollen germination rank was represented by ranks with area of pollinia stained: (0) no-germination; (1) 1–20%; (2) 21–40%; (3) 41–60%; (4) 61–80%; (5) 81–100%

Pollinia of *Phalaenopsis* Little Gem Stripes at 6 stages of flower development were harvested for comparison of in vitro pollen germination ability (Fig. 1A) and a proper stage was chosen for storage experiment. Flower development stages were defined as: (1) floral bud; (2) sepal half-open; (3) flower half-open; (4) prior to anthesis; (5) flower fully open; (6) 1 day after fully open.

Before storage, pollinia were kept at room temperature for 11 days until constant weight. Air-dried pollinia from flower stage 5 which revealed highest in vitro germination rate were wrapped with aluminum foil, sealed and packed into zip-lock bags, and stored at room temperature (RT), 4, − 20, and − 80 °C, respectively. Pollinia were sampled for both viability assessment and pollination tests after 0, 4, 8, 16, 24, 32, 40, 48, 56, 64, 72, 80, 88, and 96 weeks of storage. The stored pollinia were pre-hydrated in reverse osmosis (RO) water at room temperature for at least 30 min before in vitro pollen germination assay.

### Pollen viability evaluated by staining

The viability of stored pollinia was assessed by staining with 2,3,5-triphenyltetrazolium chloride (TTC, Sigma Aldrich, USA) (Matison et al. 1947; Khatun and Flowers 1995; Abdelgadir et al. 2012). The pollinia were placed in a 1.5 ml centrifuge tube containing 0.5% TTC aqueous solution and incubated for 24 h at 25 ± 1 °C in darkness. The 0.5% TTC aqueous solution was prepared by dissolving 0.5 g TTC in 50 mM sodium phosphate buffer solution, pH 7.4. The stained pollinia were rinsed three times with RO water to remove extra stain. The stained pollinia were examined under a stereo microscope (Stemi SV6 Zeiss, Germany). Area of pollinia stained red was considered viable, while the unstained pollinia portion were considered non-viable.

### In vitro pollen germination

Pollens taken from part of pollinia were dipped in 70% alcohol for surface sterilization about 10 s and cultured in a germination medium containing 100 mg l^−1^ boric acid, 300 mg l^−1^ calcium nitrate, 200 mg l^−1^ magnesium sulphate, and 100 mg l^−1^ potassium nitrate, pH 5.6, with 5% sucrose on the slides (Brewbaker and Kwack 1963). Pollinia was incubated at 25 ± 1 °C with three replications for each storage condition, and kept in the dark for 7 days. The Alexander’s stain (Alexander 1969) was prepared by mixing 10 ml 95% ethanol, 10 ml Malachite green (1% solution in 95% ethanol), 50 ml distilled water, 25 ml glycerol, 5 ml acid fuchsin (1% solution in water), 0.5 ml orange G (1% solution in water), 4 ml glacial acetic acid and volume adjusted to 100 ml by RO water. Pollens were carefully squashed in the dye solution as evenly as possible, and pollen tube was visualized under a microscope at 100× magnification and about 200 pollen grains of each replication were observed. Pollen grains were considered to be germinated when the pollen tube elongated to twice the pollen grain size. Level of pollen germination was represented by ranking: 0, no-germination; 1, 1–20%; 2, 21–40%; 3, 41–60%; 4, 61–80%; 5, 81–100% germination.

### Hand pollination and observation of in vivo pollen tubes

The stored pollinia at different period were removed and hand pollinated to the female parent *Phalaenopsis* Sogo Vivien ‘F858’ (Sogo Orchid Nursery, Pingtung, Taiwan). The female plants were grown in the Pad and Fan greenhouse at National Pingtung University Science and Technology (Pingtung, Taiwan), which received a photosynthetic photon flux of 346.4 μmol m^−2^ s^−1^ in summer at noon. Plants were fertilized weekly with 0.5 g l^−1^ of Peters^®^ 20 N-20P_2_O_5_-20K_2_O solution (Scotts, USA). Four flowers were pollinated per storage temperature condition. The capsule setting was recorded 4 months after successful pollination. To examine pollen tube growth, parts of the capsule (ovary) were excised at 7, 35, and 70 days after pollination. The ovary tissues were fixed in FAA (formalin: acetic acid: 95% ethanol = 1:1:18) overnight, and washed three times by distilled water, and stored in 70% ethanol. The ovaries were softened in a 4 N sodium hydroxide solution at 60 °C and stained by use of a buffer solution containing 0.1% water-soluble aniline blue dye dissolved in 0.1 N K_3_PO_4_. The ovary tissue was carefully squashed on a slide and observed under a microscope supplied by ultraviolet light of a wavelength at 356 nm (Martin 1959). Finally, the mature capsules were harvested after 4.5 months and the amount of seed was estimated visually. Level of seed amount was represented by ranking: lacking or no any seed (−), low abundance (+), abundant (++), and very abundant (+++), respectively. The presence of embryos in the seeds was examined under a light microscope (Olympus BX50F-3, Japan) by staining with 1% acetocarmine before observation. The seeds were then sterilized with 0.6% sodium hypochlorite solution for 10 min then washed 3 times with sterile water. Later the sterilized seeds were sown on a germination medium containing Hyponex (7N-6P_2_O_5_-19K_2_O) plus 1 g l^−1^ Bacto-tryptone, 50 g l^−1^ potato homogenate, 50 g l^−1^ banana homogenate, 30 g l^−1^ sucrose, 2 g l^−1^ activated charcoal, and 7.5 g l^−1^ agar as reported previously (Huang et al. 2014).

### Statistical analysis

The experimental design for pollen germination was completely randomized design (CRD) with three replications for each storage condition. Data were analyzed by using analysis of variance (ANOVA) offered in SAS version 9.0 (SAS Institute Inc., Cary, NC, USA) and mean separation was compared by using Least Significance Difference test (LSD) (p ≤ 0.05).

## Results

### Germination of pollen from different stages of flower development

Pollinia were taken from 6 flower development stages. The in vitro germination rate increased gradually along with the flower stages (Fig. 1A, B). The pollen of first flower stage already showed germination ability. Pollen at fifth flower stage showed highest germination rate followed by the sixth stage. The observation here indicated that fifth-stage probably was the best for pollen storage.

### Pollen storage capability at different temperatures

**Fig. 2:**
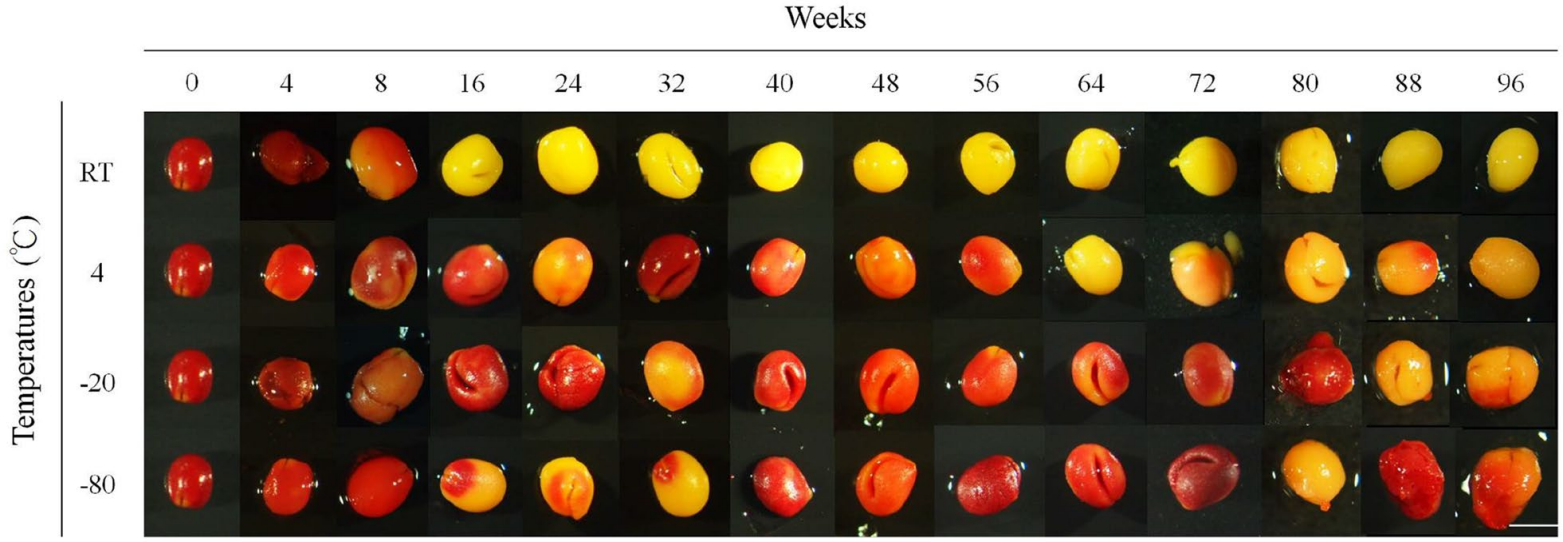
The pollen viability of *Phalaenopsis* Little Gem Stripes stored at different temperatures as evaluated by TTC staining. RT, room temperature. Scale bar = 5 mm

Freshly-collected pollinia from stage 5 flower showed prominent viability as revealed by heavy TTC staining (Fig. 2). The stained area on the pollinia represented different degree of viability, with yellow or un-stained area being considered non-viable or low viability (Fig. 2).

**Fig. 3:**
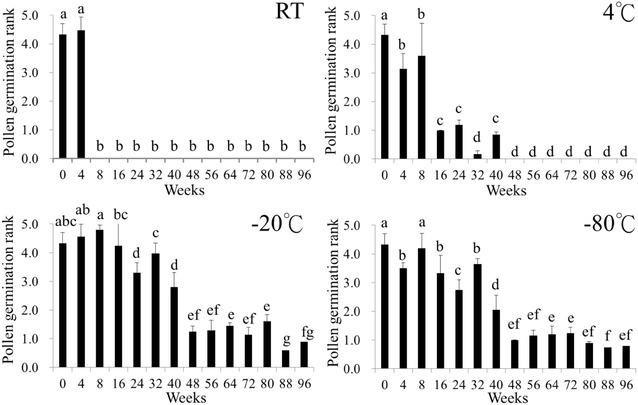
Pollen germination rate of *Phalaenopsis* Little Gem Stripes after storage at four temperatures. In vitro pollen germination of *P.* Little Gem Stripes was evaluated by incubating pollinia in Brewbaker and Kwack medium after 7 days and then stained. Bars represent mean ± SE, n = 4. Pollen germination rank was represented by ranks: (0) no-germination; (1) 1–20%; (2) 21–40%; (3) 41–60%; (4) 61–80%; (5) 81–100%

Next, we examined the in vitro and in vivo germination of stored pollen and the control, the freshly-collected pollen of *P.* Little Gem Stripes. The germination rate of fresh pollen was significantly higher and those stored at all temperatures for 4 weeks were also similar (Fig. 3). Pollen stored at room temperature beyond 4 weeks drastically lost its viability. Pollen germination at lower storage temperatures remained at the reasonable rate but gradually declined with increasing storage period. At 4 °C for 32–40 weeks, germination rate was reduced drastically and showed much lower viability. While the germination rate still remained about the same level as at 4 °C for 40 weeks when stored for 96 weeks at both − 20 or − 80 °C.

**Fig. 4:**
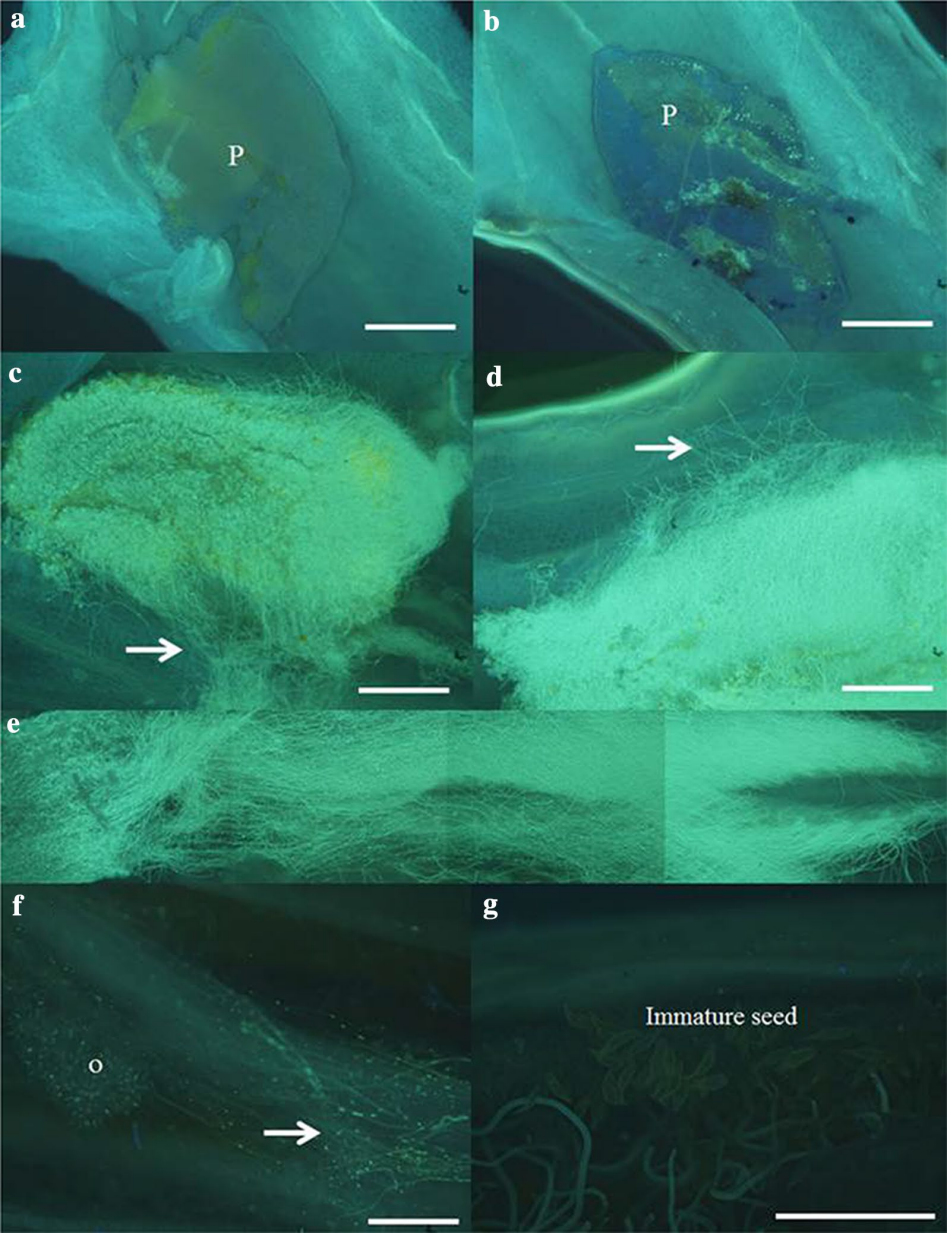
Pollen tubes of *Phalaenopsis* Little Gem Stripes grown in stigma and ovary cavity of *P*. Sogo Vivien ‘F858’ after storage. **a**–**d** The pollen taken from storage for 96 weeks at four temperatures was pollinated for 7 days. **a** room temperature storage; **b** 4 °C; **c** − 20 °C; **d** − 80 °C. **e** Pollen from storage at − 20 °C was pollinated for 35 days, and **f** for 70 days, **g** partially magnified from **e**. Arrow indicates pollen tube (**c**, **d**, and **f**). P, pollinia; O, ovules. Scale bars = 50 μm

To further examine the effectiveness of stored pollen at all period, we pollinated it to a female parent *P*. Sogo Vivien ‘F858’ and then checked pollen tube growth after 7–70 days (Fig. 4). No pollen tube growth was observed when stored at room temperature and at 4 °C after 96 weeks (Fig. 4a, b). Pollen stored at both − 20 and −80 °C after 96 weeks exhibited pollen tube growth into stigma at 7 days (Fig. 4c, d) and at 35 days (Fig. 4e) and 70 days (Fig. 4f). Immature seeds were observed after 70 days (Fig. 4g) and viable seeds were obtained 4.5 months after pollination. The seeds could be germinated in vitro readily in a germination medium as reported previously (Huang et al. 2014). Since the germination of all examined capsules was all evenly and later developed into seedlings, therefore we did not perform statistical analysis.

The pollen stored at room temperature beyond 4 weeks did not show any viability (Fig. 3) and failed to produce any capsule after pollination, while those stored at lower temperatures resulted in successful fruit set (Table 1). At 4 °C for 56 weeks, 25% capsule set was obtained. When pollen stored at both −20 and − 80 °C after 96 weeks, 50% fruit set was still recorded (Table 1). The mature capsules were harvested after 4.5 months and the relative amount of seeds decreased when the pollen was stored for a longer period (Table 2).

**Table 1:** Percentage of capsule set after pollination of stored pollinia at four temperatures

	Storage weeks													
	0	4	8	16	24	32	40	48	56	64	72	80	88	96
RT	100^a^	100	0	0	0	0	0	0	0	0	0	0	0	0
4 °C	100	100	100	100	100	75	50	50	25	0	0	0	0	0
− 20 °C	100	100	100	100	100	100	100	100	100	50	25	25	25	50
− 80 °C	100	100	100	100	100	100	75	100	100	75	100	100	75	75

RT, room temperature

^a^*P.* Sogo Vivien ‘F858’ as female parent. Successful pollination percentages recoded after 4 months. n = 4

**Table 2:** The amount of seeds in mature capsule after hand pollination of stored pollinia at four temperatures

	Storage weeks													
	0	4	8	16	24	32	40	48	56	64	72	80	88	96
RT	+++^a^	++	ND	ND	ND	ND	ND	ND	ND	ND	ND	ND	ND	ND
4 °C	+++	+++	+++	+++	++	++	+	−	−	ND	ND	ND	ND	ND
− 20 °C	+++	+++	++	+++	+++	+++	++	+	++	+	+	+	−	+
− 80 °C	+++	+++	++	++	++	++	++	+	+	+	+	+	+	+

RT, room temperature

^a^ND, not determined (the capsules failed to develop for 7 days after pollination); seeds content per capsule: lacking (−), low (+), abundant (++), and very abundant (+++), respectively

## Discussion

Fresh pollen at different stages of flower development showed germination ability with various degrees. Our previous study on *Doritaenopsis* meiosis revealed matured pollens finished meiosis and were kept at tetrad stage that was ready for germination after pollination (Bolaños-Villegas et al. 2008). In cherimoya, pollen exhibited the highest germination rate at anther dehiscence than either at 30 and 5 h before dehiscence or at 20 h after dehiscence (Rosell et al. 2006). Wang et al. (2015) observed that the pollen in litchi showed the better quantity in the filaments extended farther before the anther cracked. Germination increased to 80% in *Anacamptis morio* at the beginning of anthesis (Marks et al. 2014), at which time the moisture of fresh pollen remains higher. The pollen germinability was affected not only by the anther dehiscence but also the pollen age (Bellusci et al. 2010; Marks et al. 2014). Our results showed the pollen of *P.* Little Gem Stripes showed the highest germinability at fifth flower development stage (Fig. 1).

The moisture content at reduced level is important in pollen longevity for long-term preservation. Pollinia weight was reduced to about 50% after 11 days of drying in our study (data not shown). In *Dactylorhiza maculate* pollen moisture of 10% showed the highest germination, however, the pollen germination decreased by increasing the moisture contents (Marks et al. 2014). Our work and others revealed that moisture content of pollen was critical before sub-zero storage. Previous reports have confirmed pollen viability when stored at lower temperature. Our result showed that partially stained pollinia were eminent beginning from 8 to 16 weeks of storage at low temperature. After 96 weeks of storage at 4, − 20 and − 80 °C, pollinia showed light-red to red stain, indicating partial viability remained by TTC staining (Fig. 2). Litchi pollen stored at 4 °C remained viable after 52 days (Wang et al. 2015). Martínez-Gómez et al. (2000) reported almond pollen could be stored at 4 °C for 8 weeks. In this study, the pollen of *P.* Little Gem Stripes stored at 4 °C for at least 40 weeks remained viable (Fig. 3). The pollen germinability stored at lower temperatures varies among plant cultivars. Pollen of four California almond cultivars, ‘Nonpareil’, ‘Ne Plus Ultra’, and ‘Sonora’, stored at 4 °C for 12 months retained 8–50% pollen germination (Martínez-Gómez et al. 2002). On the other hand, pollen of *Protea repens*, *P. magnifica*, *P. eximia*, and *P. aristata* stored at 4 °C could be germinated after storage for 270, 90, 90, and 30 days, respectively, and retained over 60% germination after 360 days (van der Walt and Littlejohn 1996). Furthermore, the pollen of *Dactylorhiza fuchsii* stored at – 20 °C for 6 years still had 64% germination (Marks et al. 2014). Pollen kept at a sub-freezing temperature was used for long-term storage of litchi (Wang et al. 2015).

The fertilizing ability rather than the viability of pollen stored at low temperature was affected (Lyakh et al. 1998; Marks et al. 2014; El-Homosany and Sayed 2015). Metz et al. (2000) observed that pollen of *Hylocereus* stored at 4 °C for 3 or 9 months exhibited only 60–70% fruit set after pollination, but storage at sub-freezing temperature for 3 or 9 months still had 100% fruit set. The pollen of *D. fuchsii* stored at – 20 °C for 6 years showed reduced viability and thus influenced seed yield after pollination and fruit setting (Marks et al. 2014). However, when the pollen of *Brassica napus* L. was stored at 3 or 10 °C, the seed number was decreased with increasing storage period (Lyakh et al. 1998). In our study, when pollen of *P.* Little Gem Stripes stored at both − 20 and − 80 °C after 96 weeks remained viable (Figs. 3, 4), and the pollination capability and amount of seeds were gradually reduced following by increasing storage period (Tables 1, 2) but was still better than those at 4 °C. Although seed yield after pollination of stored pollens of *D. fuchsii* were reduced with increasing storage time, the germination of seed was not affected (Marks et al. 2014). In our study, the mature seeds were successfully obtained 4.5 months after pollination of pollinia stored at subzero temperatures to a receptive parent *P*. Sogo Vivien ‘F858’ (data not shown).

## Conclusion

This study of subfreezing storage of *Phalaenopsis* pollinia demonstrated the possibility of long-term preservation of at least 96 weeks. For simplicity of viability determination after subfreezing storage, hand-pollination and seed set probably is the best measure in *Phalaenopsis* breeding program for easy operation.

## References

[CR1] Abdelgadir HA, Johnson SD, Van Staden J (2012). Pollen viability, pollen germination and pollen tube growth in the biofuel seed crop *Jatropha curcas* (Euphorbiaceae). S Afr J Bot.

[CR2] Ajeeshkumar S, Decruse SW (2013). Fertilizing ability of cryopreserved pollinia of *Luisia macroantha*, an endemic orchid of Western Ghast. Cryo Lett.

[CR3] Alexander MP (1969). Differential staining of aborted and nonaborted pollen. Stain Technol.

[CR4] Bellusci F, Musacchio A, Stabile R, Pellegrino G (2010). Differences in pollen viability in relation to different deceptive pollination strategies in Mediterranean orchids. Ann Bot.

[CR5] Bolaños-Villegas P, Chin SW, Chen FC (2008). Meiotic chromosome behavior and capsule setting in *Doritaenopsis* hybrids. J Amer Soc Hort Sci.

[CR6] Bou Daher F, Chebli Y, Geitmann A (2009). Optimization of conditions for germination of cold-stored *Arabidopsis thaliana* pollen. Plant Cell Rep.

[CR7] Brewbaker JL, Kwack BH (1963). The essential role of calcium ion in pollen germination and pollen tube growth. Am J Bot.

[CR8] Christenson EA (2001). Phalaenopsis: a monograph.

[CR9] Council of Agriculture, Executive Yuan (2016) Agricultural statistic information (2016). http://agrstat.coa.gov.tw/sdweb/public/trade/tradereport.aspx. Accessed 29 Mar 2017

[CR10] de Souza EH, Souza FVD, Rossi ML, Brancalleão N, da Silva Ledo CA, Martinelli AP (2014). Viability, storage and ultrastructure analysis of *Aechmea bicolor* (Bromeliaceae) pollen grains, an endemic species to the Atlantic forest. Euphytica.

[CR11] Deng Z, Harbaugh BK (2004). Technique for in vitro pollen germination and short-term pollen storage in Caladium. HortScience.

[CR12] Dutta SK, Srivastav M, Chaudhary R, Lal K, Patil P, Singh SK, Singh AK (2013). Low temperature storage of mango (*Mangifera inidica* L.) pollen. Sci Hort.

[CR13] El-Homosany AA, Sayed HA (2015). Effect of low temperature and cryopreservation on in vitro pollen germination of some olive cultivars. Am Euras J Agric Environ Sci.

[CR14] Galleta G, Moore J, Janick J (1983). Pollen and seed management. Methods in fruit breeding.

[CR15] Heslop-Harrison J, Heslop-Harrison Y (1970). Evaluation of pollen viability by enzymatically induced fluorescence; intracellular hydrolysis of fluorescein diacetate. Biotechnol Histochem.

[CR16] Huang YW, Tsai YJ, Cheng TC, Chen JJ, Chen FC (2014). Physical wounding and ethylene-stimulated embryogenic stem cell proliferation and plantlet regeneration in protocorm-like bodies of *Phalaenopsis* orchids. Genet Mol Res.

[CR17] Khatun S, Flowers TJ (1995). The estimation of pollen viability in rice. J Exp Bot.

[CR18] Lora J, Pérez de Oteyza MA, Fuentetaja P, Hormaza JI (2006). Low temperature storage and in vitro germination of cherimoya (*Annona cherimola* Mill.) pollen. Sci Hort.

[CR19] Lyakh VA, Soroka AI, Kalinova MG (1998). Pollen storage at low temperature as a procedure for the improvement of cold tolerance in spring rape, *Brassica napus* L. Plant Breed.

[CR20] Marks TR, Seaton PT, Pritchard HW (2014). Desiccation tolerance, longevity and seed-siring ability of entomophilous pollen from UK native orchid species. Ann Bot.

[CR21] Martin FW (1959). Staining and observing pollen tubes in the style by means of fluorescence. Biotechnol Histochem.

[CR22] Martínez-Gómez P, Gradziel TM, Ortega E, Dicenta F (2000). Short-term storage of almond pollen. HortScience.

[CR23] Martínez-Gómez P, Gradziel TM, Ortega E, Dicenta F (2002). Low temperature storage of almond pollen. HortScience.

[CR24] Masum-Akond ASMG, Pounders CT, Blythe EK, Wang XW (2012). Longevity of crapemyrtle pollen stored at different temperatures. Sci Hort.

[CR25] Matison AM, Jensen CO, Dutcher RA (1947). Triphenyltetrazolium chloride as a dye for vital tissues. Science.

[CR26] Metz C, Nerd A, Mizrahi Y (2000). Viability of pollen of two fruit crop cacti of the genus *Hylocereus* is affected by temperature and duration of storage. HortScience.

[CR27] Parton E, Vervaeke I, Delen R, Vandenbussche B, Deroose R, De Proft M (2002). Viability and storage of bromeliad pollen. Euphytica.

[CR28] Peng H-Z, Jin Q-Y, Ye H-L, Zhu T-J (2015). A novel in vitro germination method revealed the influence of environmental variance on the pecan pollen viability. Sci Hort.

[CR29] Pritchard HW, Prendergast FG, Pritchard HW (1989). Factors influencing the germination and storage characteristics of orchid pollen. Modern methods in orchid conservation: the role of physiology, ecology, and management.

[CR30] Rosell P, Saúco VG, Herrero M (2006). Pollen germination as affected by pollen age in cherimoya. Sci Hort.

[CR31] Soares TL, Silva SO, Costa MAPC, Santos-Serejo JA, Souza AS, Lino LSM, Souza EH, Jesus ON (2008). In vitro germination and viability of pollen grains of banana diploids. Crop Breed Appl Biotechnol.

[CR32] Sorkheh K, Shiran B, Rouhi V, Khodambashi M (2011). Influence of temperature on the in vitro pollen germination and pollen tube growth of various native Iranian almonds (*Prunus* L. spp.) species. Trees.

[CR33] Sweet HR (1980). The genus *Phalenopsis*.

[CR34] Vaknin Y, Disikowitch D (2000). Effects of short-term storage on germinability of pistachio pollen. Plant Breed.

[CR35] Vaknin Y, Mills D, Benzioni A (2003). Pollen production and pollen viability in male jojoba plants. Ind Crop Prod.

[CR36] van der Walt ID, Littlejohn GM (1996). Storage and viability testing of *Protea* pollen. J Amer Soc Hort Sci.

[CR37] Wang M-L, Hsu C-M, Chang L-C, Wang C-S, Su T-H, Huang Y-JJ, Jiang L, Jauh G-Y (2004). Gene expression profiles of cold-stored and fresh pollen to investigate pollen germination and growth. Plant Cell Physiol.

[CR38] Wang L-M, Wu J-F, Chen J-Z, Fu D-W, Zhang C-Y, Cai C-H, Ou L-G (2015). A simple pollen collection, dehydration, and long-term storage method for litchi (*Litchi chinensis* Sonn.). Sci Hort.

